# Superstition in Medicine

**Published:** 1906-03

**Authors:** 


### BOOK REVIEWS.

Superstition in Medicine. By Professor Dr. Hugo Magnus. Authorized translation from the German by Dr. Julius L. Salinger. 12 mo. pp. 205. [New York and London: Funk & Wagnalls Company.] 1905. Price, $1.00 net.

This is certainly an interesting contribution to the history of medicine which should be read by the medical and dental profession alike. Whilst very amusing in certain portions it is instructive as well and when read between the lines, gives much food for thought. While the book may appear a small one it has involved much labor and research on the part of the author to find the authorities to consult and thus make the book a reliable and an authoritative one. A reference to the bibliography which is appended to the text will amply demonstrate the labor which was necessitated as well as the expenditure of time.

The book is well worth the price asked for it and our only regret is that the author did not see fit to make it more voluminous. The translator has acquitted himself well of his task and the additional chapter from his pen is excellent.

